# Experiences of patients having tuberculosis (TB) regarding the use of Directly Observed Treatment Short-Course (DOTS) in the North West Province, South Africa

**DOI:** 10.4102/curationis.v39i1.1629

**Published:** 2016-09-27

**Authors:** Maserapelo G. Serapelwane, Mashudu Davhana–Maselesele, Gaboipolelwe M. Masilo

**Affiliations:** 1 Department of Nursing, North West University, Mafikeng Campus, South Africa

## Abstract

**Background:**

Tuberculosis (TB) management remains a major challenge despite the implementation of Directly Observed Treatment Short-Course (DOTS). Some of the challenges include defaulting treatment, low TB cure rates and relapse after patients had been treated under DOTS.

**Objectives:**

This study explored and described experiences of patients having TB regarding the use of DOTS in Doctor Ruth Segomotsi Mompati District of North West Province, South Africa. The study describes and recommends support required by patients having TB who are using DOTS.

**Methods:**

A qualitative, exploratory, descriptive and contextual design was used. The population consisted of all patients having TB under DOTS who had taken treatment for 2 months and more in one of the community health centres in Doctor Ruth Segomotsi Mompati District. Purposive sampling technique was applied to select participants receiving DOTS service. In-depth unstructured individual interviews were conducted, and data saturation occurred after having interviewed 15 participants. Ethical considerations were ensured throughout the study, and data were analysed using Tesch’s method of coding and analysis.

**Results:**

Two themes emerged from data and these are discussed as concerns related to ineffective use of DOTS and lack of resources as contributory factor to ineffective use of DOTS. Among other categories, poor nurse–patient relationships and difficulties in accessing the community health centre emerged as consistent themes related to default and inconsistent use of DOTS.

**Conclusion:**

Ineffective use of DOTS contributed to TB treatment default and low cure rate. Therefore, recommendations focused on strengthening effective use of DOTS for the management of TB.

## Introduction and background

Tuberculosis (TB) remains a major global health problem. The literature revealed an estimated 8.6 million people have developed TB and 1.3 million have died from the disease (World Health Organization 2013:8). In 1993, TB was declared a global emergency by the World Health Organization (WHO) and the organisation began promoting a management and patient support strategy known as Directly Observed Treatment Short-Course (DOTS) (Out 2013:1). When using DOTS, anti-TB medications are taken by patients under the supervision of a health worker. DOTS ensures that proper medications are given at proper intervals and at the right doses (Out 2013:1). By 2005, 187 countries had started implementing DOTS with 4.9 million cases of TB being treated using this strategy in that year (Out 2013:1). The reports for that year suggested that implementation of DOTS is a global success in the prevention of the spread of TB.

The study conducted in the United States by Glassman and Fan (2014:5) revealed that the best way of controlling the spread of TB is by fully supporting patients having TB by using DOTS. A similar phenomenon was reported by Biadglegne *et al*. (2013:1) as it was found that the treatment plans that emphasised support by DOTS showed improved TB cure rates. On the other hand, the history of previous default of TB treatment was associated with a lack of support by family members (Garrido *et al*. 2012:3). The findings demonstrate that support of patients having TB by using DOTS can prevent the spread of TB. The findings further reveal that the use of DOTS could remedy the challenge of low TB cure rate and treatment default.

The report revealed that patients having TB who were registered for DOTS programmes in Russia and the Philippines had better treatment outcomes than other countries in Sub-Saharan Africa. The cure rates in Russia and Philippines increased annually, and the target was reached in 2003 until 2007 (WHO 2009:12). The findings suggest that improved cure rate is one of the benefits of effective implementation of DOTS. The burden of TB is high in Asia and Africa (WHO 2012). India and China together account for almost 40% of the world’s TB cases, whereas approximately 60% of cases are in South Asia and Western Pacific regions (WHO 2012:1). These statistics clearly show that Africa is the one continent that is experiencing the highest numbers of TB cases.

Even though the benefits of using DOTS programmes in TB management have been reported, challenges regarding TB management and DOTS are also raised, especially in the developing countries. The study conducted in Ethiopia on DOTS reported that low TB cure rate was associated with poor observation of patients during the course of treatment and poor patient treatment compliance (Sisay *et al*. 2014:7).

South Africa is identified as one of the 22 high-burden countries that contribute approximately 80% of the total global burden of TB cases as reported by the TB strategic plan for South Africa (2007–2011:12). In response to the burden of TB, South Africa aligned TB management guidelines to international strategies such as Stop TB strategy and policy paper (DOH 2015:89). The goal of TB strategy and policy paper is to dramatically reduce the burden of TB in line with the 2015 global target. One of the components of Stop TB strategy is to pursue high-quality DOTS expansion and enhancement. The national development plan is another strategy in South Africa that prioritises improvement in achievement of the goals of TB by 2030. In South Africa, DOTS has long been implemented in 1997 with the aim of alleviating challenges regarding TB management (Churchyard *et al*. 2014:245). The recent study conducted in South Africa revealed three provinces with the cure rate of less than 70% against the WHO target of 85% (DOH 2015:89). Among these three provinces, North West is found to have a high defaulter rate of 7.5% against the national target of below 5% (DOH 2015:89). The high defaulter rate and low cure demonstrates that patients having TB on DOTS in the North West Province need to be listened to as they are the recipients of care. The study conducted in Bojanala health district in the North West Province revealed low TB cure rate of 47%. The same study reported that some patients missed taking treatment for some days (Tumbo & Ogunbanjo 2011:3). The TB outcome report revealed Doctor Ruth Segomotsi Mompati District in the North West Province to have a low cure rate of less than 50% (WHO 2012:1). The findings reveal the districts of the North West Province as having challenges regarding TB management. The patients are defaulting treatment, and the cure rates remain below the target in the North West Province. Hence, this study deemed it necessary to explore the experiences of patients having TB using DOTS in Doctor Ruth Segomotsi Mompati District. In addition, there are limited published studies that have explored the experiences of patients regarding the use of DOTS in the North West Province.

The study conducted in Moses Kotane Hospital in the North West Province focused on nurses’ views regarding the discharge plan of patients having TB (Motsomane & Peu 2008:3). This study insists that it is imperative to explore and describe experiences of patients having TB using DOTS as the information could assist in understanding the problems related to the use of DOTS.

### Problem statement

Despite the implementation of DOTS, the anecdotal evidence revealed challenges regarding TB management in Doctor Ruth Segomotsi Mompati of North West Province. This was observed by the researcher when she was working as a professional nurse in the primary healthcare facilities in Doctor Ruth Segomotsi Mompati District. The researcher observed patients defaulting TB treatment and low TB cure rate and relapse after patients had been treated under DOTS. The patients in whom TB relapsed were at risk of developing Multiple Drug Resistant and Extreme Drug Resistant TB. In addition, there is limited information regarding experiences of TB using DOTS in the North West Province. Thus, the following are the research questions of this study: What are the experiences of patients having TB regarding the use of DOTS and what support do they expect in order to enhance the efficient use of the DOTS programme?

### Purpose of the study

The purpose of this study was to describe and explore the experiences of patients having TB regarding the use of the DOTS programme in Doctor Ruth Segomotsi Mompati District. The study also recommended the kind of support needed for patients using DOTS in this district.

### Research objectives

The objectives of this study were to:

Explore and describe the experiences of patients having TB using DOTS, and

Describe the support required by patients having TB using DOTS.

### Meta-theoretical assumptions

According to Polit and Beck (2004:711), an assumption is a basic principle that is accepted as being true, based on logic or reason, but without proof or verification. The researcher in this study assumes that a patient is a human being who needs support, care and respect and that each patient requires a unique treatment.

### Theoretical assumptions

This study was guided by the assumptions in Watson’s theory of human caring developed in 1975 (Sisca 1989: 229). According to Watson’s theory, as in Sisca (1989:229), a helping, trusting and caring human relationship evolves from contextually modulated communication. It is assumed that communication is a reciprocal and dialogic process during which the speaker anticipates a response appropriate to the message encoded. This study assumes that good communication should be maintained between the patient, the nurse and the doctor in the primary healthcare facility and the hospital. Good communication can improve compliance to treatment when it is consistent when caring for a patient having TB on DOTS. According to Watson’s theory (in Sisca 1989:229), a focus on feeling and non-rational emotional aspects of an event are essential practical expectations for nurses, doctors and the patients engaged in the caring process.

### Definition of key concepts

**Directly Observed Treatment Short-Course (DOTS):** In this study, directly observed treatment strategy means a patient is taking treatment under supervision of a known treatment supporter.

**An adult patient having TB:** In this study, the age of an adult patient having TB ranges from 18 to 55 years.

#### Research design and method

A qualitative, descriptive, exploratory and contextual research design (Brink, van der Walt & van Rensburg 2012:11) was adapted aiming at describing the experiences of patients having TB regarding the use of DOTS in Doctor Ruth Segomotsi Mompati District.

### Population

The population of this study comprised patients having TB on DOTS at one of the community health centres in Kagisano and Molopo Sub-district in Doctor Ruth Segomotsi Mompati District of North West Province, South Africa.

### Sampling

Purposive sampling (Brink *et al*. 2012:215) was used and the focus was on patients having TB on DOTS who were registered in one of the health centres in the Kagisano Molopo Sub-district. The patients included in this study were those who were on TB treatment for 2 months or longer. This study excluded patients having TB who were newly diagnosed and had been on TB treatment for less than 2 months. The age of the participants ranged from 18 to 55 years. Both genders, men and women, were selected so that data would be representative of both sexes. The number of participants was governed by data saturation, and data collection stopped when the data saturation occurred after having interviewed 15 participants.

### Data collection methods

This study took place during October 2011. Data were collected by means of unstructured in-depth individual interviews (Brink *et al*. 2012:158) and the interviews were conducted in Setswana. Permission was requested from and granted by the participants to use an audiotape for purposes of accurate transcription afterwards. The interviews lasted between 45 and 60 minutes, and the field notes for each interview were collated for themes and patterns in the data.

### Data analysis

The study used eight steps of Tesch’s method (Creswell 2009:184) of analysis and open coding process to analyse raw data. This included organising and preparing data by making verbatim transcription of unstructured interviews and typing field notes. The researcher read and re-read through all the transcribed interviews and field notes in order to fully understand the information provided by patients having TB regarding the use of DOTS. The researcher picked one document of an interview to read and write down thoughts in the margin. This task of reading and writing thoughts in the margin was continued with other interview data gleaned from participants. The researcher generated a list of all topics that emerged and narrowed down topics into codes. Finally, a decision was reached regarding codes, including a description of themes and interpretation of the findings.

## Ethical considerations

Ethical clearance was obtained from the Ethics Committee of North-West University (NWU 00249 11 A9) and permission was granted by the North West Province Department of Health. Furthermore, permission to conduct the research was requested in writing to the manager of the district where the study took place. Informed consent was obtained from the participants in order to allow them to decide voluntarily whether to participate in the study. This was achieved by explaining the research topic, aim and objectives of the study and the purpose of the study. It was further explained to the participants that codes instead of names would appear on the interview documents to ensure anonymity. Thereafter, voluntary participation was ensured by patients having TB signing an informed consent form (Brink *et al*. 2012:36).

## Trustworthiness

The model proposed by Lincoln and Guba (1981) was used to ensure trustworthiness of the findings and criteria used were the truth value, applicability, consistency and neutrality. The strategies used to validate these four criteria were credibility, transferability, dependability and confirmability (Krefting 1991:215). The researcher had a prolonged engagement with participants and the interviews lasted 45–60 minutes. In this study, both research supervisors conducted an independent cross-check of the interview materials and transcripts to ensure that the researcher maintained consistency in asking questions and addressing the aims and objectives of this study. Again re-coding of data was done by supervisors to ensure trustworthiness of the findings.

## Research findings and discussion

Two broad themes, categories and sub-categories emerged from data. Table 1 provides a summary of findings according to themes.

**TABLE 1:** Summary of findings.

Themes	Categories	Sub-categories
Ineffective use of DOTS	Lack of communication among different stakeholders	Lack of communication between hospital doctors and clinic nurses Lack of communication between clinic nurses and patients
	Poor nurse–patient relationship	Quarrels Hurt feelings
	Lack of health education for patients using DOTS	Lack of knowledge of TB treatment phases and side effects Need for relevant health talks with patients having TB
	Lack of supervision	Lack of supervision at the clinic Lack of supervision at home
Lack of resources to implement DOTS effectively	Lack of food provisions to meet basic needs	Lack of food supplements Difficulties in accessing the clinic

*Source:* Author’s own work

### Ineffective use of DOTS

The 15 participants expressed their concerns related to ineffective use of DOTS. The categories that emerged from this theme were (1) lack of communication among different stakeholders, (2) poor nurse–patient relationship, (3) lack of health education for patients using DOTS and (4) lack of supervision. The categories are subsequently described in detail.

#### Lack of communication between hospital doctors, nurses and patients on DOTS

The participants experienced some lack of communication between hospital doctors and clinic nurses as well as between clinic nurses and patients on DOTS. This is discussed as follows:

#### Lack of communication between hospital doctors and clinic nurses caring for patients on DOTS

Nurses at the clinic wanted patients to be given a pink referral form but the doctors provided patients with an ordinary letter or a white or green card as a way of communication. It was found that nurses became angry with patients who were not given the correct referral documents by the doctors from the hospital. One of the participants said:

‘My first day on arrival for registration at the clinic from… from the hospital where I was… was diagnosed TB, I experienced some problems. I was given a letter by the doctor for me to give it to the sisters at clinic. I was sent back to hospital because the nurses said that I am supposed to come with the TB letter and at the hospital I was sent back again and the doctor said… said they… they should put me in a clinic list. The nurses again refused at TB … at TB clinic. I went back again to the hospital, then the doctor and nurses spoke telephonically, I was then was put on the clinic list.… I went back to hospital the doctor wrote another letter for me again and after that they did send [*sic*] me back again saying that they want a pink paper from the hospital and I tried to explain to them that I don’t have money for going back to hospital… I borrowed money from the neighbours [*silent and crying*].’ (Participant 5, Female, Age 28, Domestic worker)

When referring a patient having TB to another health facility, a TB ‘transfer and move’ form should be duly completed and filled in triplicate with the correct advisory information and a pink copy must be given to the patient (National Tuberculosis Control Programme 2008:8), ensuring continuation of treatment module. In this study, the patients having TB on DOTS from hospital were not given the correct ‘transfer and move’ forms, which are also called referral documents. This indicates that poor communication occurred among doctors and nurses who were caring for patients on DOTS. The poor communication resulted in the patient on DOTS being sent back and forth between the hospital and clinic.

#### Lack of communication between clinic nurses and patients on DOTS

During data collection, participants indicated that communication in their day-to-day visits to the clinic was lacking. One of the 15 participants said:

‘When I arrive at the clinic for daily treatment, nurses do not ask you any questions … you just enter … enter in the TB room to take tablet, you take your treatment and after drinking … the nurses make a tick in the green card provided to show that you came for the tablets and after that you leave, and at times they don’t tick, if they have not ticked when getting home I make a tick.’ (Participant 7, Female, Age 23, Unemployed)

The same participant said: [Silent] ‘nurses should be friendly and… be free to TB patients so that patients should take treatment as required’.

In this study, most of the participants reported that when they arrived at the clinic, they simply take the tablets placed on the table and the nurses would tick their cards, and they also noted that occasionally the ticking was not done. These findings suggest that patients took treatment at the clinic on a daily basis without the anticipated supervision from health professionals. In a study by Zvavamwe and Ehlers (2009:306), it was reported that regular supervision and feedback to patients are critical factors that motivate the maintenance of required behaviour in patients having TB.

### The poor nurse–patient relationship

A poor nurse–patient relationship emerged in the form of quarrels and deliberate hurt of patients’ feelings.

#### Quarrels

In this study, some participants reported quarrels and one said:

‘[*Frowning face*] We had a quarrel, the quarrel started when she was injecting me [*sic*], I… I … I don’t know it was so painful then I said to her: it is so painful then she [*referring to the nurse*] said… **kgaa-**[*irritated, annoyed and angry*] then I said, sister are you saying **[*kgaa*]**, I can take further steps against you, why are you not saying sorry?’ (Participant 1, Female, Age 22, Unemployed)

The study revealed a quarrel with a patient during administration of injection. Another participant said:

‘The nurses should learn to talk nicely with a person, that’s my request [*looks sad*]. There are other people who are short-tempered and at times when you talk to such people they will react by leaving you and they will stay at home. My other request is that nurses should encourage people who are taking TB treatment.’ (Participant 3, Female, Age 22, Unemployed)

This shows that patients might default treatment when they are not motivated by healthcare providers.

Njozing, Edin and Hurtig (2010:31) point out that patients having TB were unable to report their grievances for fear of retribution, where on a recurrent visit such patients would be snubbed by the nurses who are meant to monitor their drug intake. In this study, three of the participants reported that they had quarrelled with nurses and that no action had been taken to avert such incidents or their recurrence afterwards.

#### Hurt feelings

The participants expressed hurt feelings and felt unhappy and unwilling to turn up for treatment the next day. One participant had this to say:

‘When I was sick and unable to walk… I missed treatment as I could not travel to the clinic and during that time I requested my kids to go to the clinic to ask for my treatment and that day my kids were not given tablets. Instead, they sent a home-based care giver to come and convince me to go to the clinic. Upon my arrival at the clinic that day it was not nice [*sic, in a low voice*]. The sister was shouting, and said to me that I am going to die… [*sic*] I am going to die if I am not taking treatment correctly… then I replied by saying that I am not going to die because of you not supporting me and should I die I will know that is because of you who failed to support me.’ (Participant 10, Female, Age 56, Unemployed)

Good therapeutic alliance is underpinned by trust, empathy and positive regard for patients under the monitoring eye of the hospital and clinic staff (Tadesse *et al*. 2013:6). In this study, nurses were not empathetic. Instead, they uttered negative words, such as telling them that they would die and the environment was totally not therapeutic. This indicates that patients were hurt by nurses during the use of DOTS.

### Lack of health education for patients having tuberculosis using DOTS

This category is discussed as lack of knowledge of TB treatment phases by patients using DOTS and lack of knowledge of the side effects of Rifafour medication, which caused late reporting of side effects.

#### Lack of knowledge of tuberculosis treatment phases on patients using DOTS

Lack of knowledge of TB treatment phases was expressed by the participants and one had this to say:

‘[*Silent*] they have not explained to me about the reasons of them changing treatment so I just assumed that they were looking at like… we coughing up sputum and it is sent [*sic*] to the laboratory. Yes, TB is attacking me for the second time. I was told that I am going to take treatment for six months.’ (Participant 2, Male, Age 34, Unemployed)

Another participant said ‘nurses who are responsible for TB patients must teach us more about TB; they should explain to us more about TB, they can teach us more and it can be better’.

The study conducted by Dolma, Adhikari, Mohapatra and Mahanta (2011:12) revealed lack of knowledge of patients on re-treatment for TB especially with regard to preventive measures and duration of treatment. This study observed that health talks related to the duration of treatment for newly diagnosed patients, re-treatment cases and the phases of treatment were not given to patients having TB. This demonstrates that patients were at risk of defaulting treatment because of a lack of knowledge of the duration of the treatment, thus contributing to low cure rate.

#### Lack of knowledge of the side effects of Rifafour which caused late reporting of side effects

Five participants suffered side effects of treatment and one had this to say:

‘By the time I started to take treatment I was having a problem of constant dizziness, I still remember that I told the nurse but…but… that day I explained to the sisters… that day after taking treatment at the clinic I explained that it is of no use because on my way home I will vomit and I requested to be given tablets to take it [*sic*] at home to enable me to lie down for a while after drinking them but the nurse said I am naughty.… Yes… the Rifafour tablets are the ones that I did not take properly, these large ones, to tell the truth I was throwing them away… it was because of vomiting afterwards. I will vomit again yes.… I think it was for about a month I threw the tablets away.… Because after that the treatment was changed and I took it properly thereafter I was given Rifinah and I had no nausea until I completed treatment.’ (Participant 2, Male, Age 34, Unemployed)

Rifafour e-200 is a combination drug for treating TB and one of the adverse side effects of this drug is nausea and vomiting because it has a metallic taste (South African Medicines Formulary 2003:300–302). It is clearly revealed that this patient was not aware that the problems she encountered were related to the side effects of Rifafour. This demonstrates that lack of knowledge of the side effects of TB drugs contributed to high treatment defaulter rate and low cure rate.

#### Need for relevant health talks with patients having tuberculosis

The need for relevant health talks with patients having TB also emerged, as one of the participants said:

‘Every Thursday we get into a meeting they teach, they are talking about the leading province that experience problems of TB. During last week they also said that this clinic [*referring to the community health centre where this study took place*] is the highest with regard to the number TB patients. According to the way we are taking treatment at the clinic on daily basis, the nurses also explained the reason to go daily to the clinic is because there are people who are throwing tablets in toilets.’ (Participant 13, Male, Age 39, Unemployed)

The participants revealed nurses giving health talks that were not clear and relevant enough to address patients’ problems concerning TB and DOTS. This demonstrates ineffective use of DOTS and a need for relevant and clear health talks with patients having TB. Watson’s theory of human caring explains that the teaching role is critical in a caring relationship. It was further revealed that caring depends on the nurses’ ability to accurately assess patients’ understanding (Sisca 1989:230).

### Lack of supervision

During discussions, it was found that supervision of patients was lacking while they took treatment. This is subsequently discussed as lack of supervision at the clinic and at home.

#### Lack of supervision at the clinic

There was no direct supervision of patients who were taking the tablets daily at the clinic and one of the participants had this to say:

‘When we arrive there we will get a lot of tablets placed on the table. The nurses do not give us tablets they just tell us that we should take and drink tablets for ourselves without them checking whether we are taking the right treatment or not, which is not the right thing to do, because other patients just drink without knowing whether this is the right or wrong tablet to take. Entlik… … [*Looks angry*] it is all the same there is no caring because even if a person is unable … very sick.… [*Jumping to another statement*] There are people who do not read when they get a lot of tablets on the table. They just take any tablets and leave with no one observing or making sure that a patient did take and drink tablets, other patients just come and leave.’ (Participant 4, Male, Age 26, Unemployed)

The same participant said:

‘If maybe someone could talk to the nurses so that they should observe patients when coming to the clinic to find out whether they are taking treatment appropriately. The nurses must be the ones who are giving patients treatment, so that when talking to someone about being cured from TB, one must say that with confidence knowing that I was given treatment by a nurse not by myself taking it from the table.’ (Participant 4, Male, Age 26, Unemployed)

This particular patient reported that different tablets were placed on the table and it was not easy for patients to pick up the correct name and strength of a prescribed drug because some patients were illiterate. The findings of this study suggest that patients having TB were exposed to non-compliance to TB treatment because of a lack of actual observation and giving patients appropriate TB drugs at the clinic. Therefore, DOTS was used ineffectively. Dooley *et al*. (2011:4) revealed in their study that non-compliance to TB treatment was associated with ineffective use of DOTS.

#### Lack of supervision at home

It was found that all patients who were using DOTS at the health centre were given treatment to take at their homes for 3 days. The patients visited the clinic from Monday to Thursday and thereafter were supplied with 3 days’ treatment.

One participant said:

‘Neeh… the nurses supply me with treatment of Friday [*sic*] until Sunday and when I got treatment from the clinic I put them in my bedroom. As I said it will be given to me… it will be given to me on Thursday so my mother sometimes she remind me [*sic*] to take tablets.’ (Participant 6, Male, Age 19, Unemployed)

The findings of this study revealed no direct observation and supervision of patients when taking treatment at home. These suggest that the patients having TB were at risk of defaulting treatment because of lack of supervision. Motsomane and Peu (2008:62) pointed out that families of patients having TB need to be educated on TB and DOTS as it improves compliance with treatment.

### Lack of resources to implement DOTS effectively

According to what was said by the participants, it was obvious that lack of resources contributed to ineffective use of DOTS. The categories that emerged from this theme are discussed as (1) lack of provision to meet the basic needs of patients having TB.

#### Lack of provision to meet the basic needs of patients having tuberculosis

The participants specified the basic challenges they encountered, which also contributed to ineffective use of DOTS. This is discussed as lack of food supplements and difficulties in accessing the clinic.

#### Lack of food supplements

During data collection, the participants reported that lack of food contributed to ineffective use of DOTS.

A participant said: ‘I feel dizziness after taking treatment because at times I go to clinic without breakfast taken … and these tablets increases one’s appetite and you will always feel hungry’.

Another one said:

‘We … you get two phuzamandla, you get two and at times one… you eat that for two days or three days then it get finished [*sic*]. You will wait for Thursday for you to get some. To tell the truth the supplement helps so much because I was unable to eat soft porridge at home. I was only eating phuzamandla so when I took it I was able to take tablets.’ (Participant 1, Female, Age 22, Unemployed)

In a study conducted by Govender and Mash (2009:514), it was found that lack of food and poverty was associated with poor adherence to TB treatment. In this study, the majority of the participants complained of hunger, suggesting that they did not take their prescriptions as indicated by the dosage. The findings of this study suggest that patients having TB need to be supported with more food for effective use of DOTS.

### Difficulties in accessing the health centre

During the interviews, it was discovered that patients were walking long distances to the health centre.

One of the participants stated:

‘I had swelling of my legs as well as abdomen and [was] unable to walk for a long distance but I endured… [*Frowning face*]. Walking… walking on daily basis to the clinic. I was walking with difficulty, moving slowly… by slipping down my feet [*silent for a moment and eyes filled with tears*]. I felt pain on my abdomen radiating to the legs. Pain caused me to have difficulty in breathing and since after taking treatment the pain is mild and.… The swelling in the abdomen subsided… but now I am much better. We need transport because we are staying too far [*two and half kilometres from the clinic*]. At times you arrive late at the clinic with so many things happening… [*Tears on face*]… alone on your way to the clinic. I request the government to help us with transport because at times you get so sick on your way to the clinic with no one to help and also with no money to pay for local transport.’ (Participant 8, Female, Age 31, Unemployed)

Another participant identified difficulty in accessing the clinic and said: ‘I am accompanied by my uncle with a donkey cart because I am unable; our place is far from the clinic. I am suffering painful legs (sic). I am only using a donkey cart, yes’.

This study clearly identified that sick patients having TB walked long distances. Some of the participants explained that they used donkey carts for accessing health facilities. Those who were staying on nearby farms reported that treatment was defaulted because of lack of money to get to the clinic. These observations confirm the findings by Govender and Mash (2009:514) that defaulting treatment was significantly associated with the distance to and from the hospital. The findings of this study clearly show that health services were not accessible to patients having TB in rural areas.

## Limitations of the study

Some of the patients who were interested and volunteered to participate were recent admissions of less than 2 months. The state of illness of other patients could not allow them to withstand interviews. Therefore, the researcher could not interview patients having TB on DOTS who had only been on the DOTS for a short time as well as those who appeared very ill but had been on DOTS for longer. The study was conducted in one Health Care Centre; thus, findings from this study could not be generalised. The study could be replicated in another setting.

## Recommendations

Based on the findings of this study, the following are the recommendations for effective use of DOTS:

### Nursing practice

#### Communication and support of patients suffering from tuberculosis on DOTS during referral

There should be effective and quality communication between doctors and nurses to improve the support and continuity of care to patients having TB. Complete filling of appropriate referral documents stated in TB guidelines should be complied with. The communication should be continued telephonically when necessary.

#### A planned referral to the clinic and to the community member enhances support of patients on DOTS

A contact person should be identified immediately so that the involvement of a treatment supporter is initiated at an early stage of treatment. Nurses at the clinic level should involve family members, and the roles of family members with regard DOTS should be clarified. The possibilities of developing side effects from DOTS drugs should be addressed on the first contact with the patient and family member(s). Community volunteers should be involved by identifying patients who will need their support. The mobile clinic staff should be involved in providing treatment to patients who stay at a distance from the clinic. The facility manager, TB coordinator and Assistant Director for Community Health Services should provide consistent support to the clinic staff for effective use of DOTS.

#### Training for multidisciplinary team involved in treating patients suffering from tuberculosis

Nurses and doctors should receive regular training regarding the management of TB at the hospital to clinic and community level. Documents related to referral from the hospital to the clinic should be included in the training to ensure that everyone who will continue with care and support of the patient is informed on how to proceed. Such training should include the implementation of DOTS. Home-based caregivers and other community workers should be included in these workshops. In-service training must be done on a regular basis for improving adherence to TB guidelines. Nurses should be trained on communication skills to improve on nurse–patient relationship.

#### Use of suggestion and complaints box

The patients having TB on DOTS should be encouraged to use a suggestion and complaints box. This could expose them to an environment where they can express their concerns without fear. The complaints in the suggestion box should be addressed on a monthly basis.

#### Provision of treatment counselling

Regular and relevant health education should be given to patients having TB, health talks should be specific and the topics should address DOTS and the importance thereof. The health education plan should also include TB treatment phases according to regimens, protocol to be followed when changing treatment dosages for patients because some participants expressed surprise during the change of treatment, especially when these patients had minor and major side effects from previous treatment.

### Supervision in the community health centre

The principles for administration of medicines should be reinforced. Supervision at the clinic should be strengthened and TB medication should be kept under lock and key, meaning that the dispensing point should be a medicine trolley or tray not an open space table. Professional nurses should be the ones to dispense TB drugs.

#### Support during early phase of treatment

The patients having TB who are staying further than 2 km away have to be attached to the nearest community volunteer. The family members of such patients should be actively involved in the support of patients having TB on DOTS.

#### Nursing education

The curriculum for basic nurse training for all levels should include management of all categories of patients having TB. This could ensure that nurses at the beginning level acquire knowledge of TB and effective use of DOTS.

#### Policy

This study recommends research that will review the national social grants policy. The policy could make provision for disadvantaged patients having TB to receive social grants at an early phase of treatment. This could alleviate situations of patients defaulting treatment because of lack of food and hunger.

#### Future research

It is important for further research to be conducted that focuses on the views of nurses who are caring for patients having TB, so as to obtain a broader view of challenges facing the DOTS programme. Research exploring support of nurses caring for patients having TB is also recommended. Equally, research on patient satisfaction with the service in rural areas needs to be conducted so as to obtain a broader view on challenges facing the health services.

## Conclusion

The qualitative exploratory and descriptive study conducted on patients having TB revealed that DOTS was used ineffectively in one of the primary healthcare facilities in the North West Province. The study revealed ineffective use of DOTS which occurred because of a lack of communication between healthcare providers at the hospital and the community health centre. The nurse-patient relationship was also a very serious concern as the patients experienced quarrels and hurt feelings. The standard of sharing information to patients having TB was found to be of low quality as irrelevant topics were discussed with patients. Nurses who were not informed about the protocol and guidelines of TB management were posing a huge challenge in the control of TB. The findings of this study revealed nursing care standards that were highly compromised as the rules of giving treatment to patients were not adhered to. By allowing patients to take treatment on their own without actually giving it to them at the health facility is a medico-legal hazard because the medicines should be handled by a trained health professional. In this study, a large number of tablets were placed on the table for patients to take. Therefore, patients did self-administration of tablets which were also not kept safe. Furthermore, very ill patients having TB were exposed to the risk of developing serious complications and possible death during their daily visits to the clinic. This indicates poor nursing administration, which caused ineffective use of DOTS strategy. This further suggests poor supervision and lack of support to nursing personnel dealing with TB. Based on the findings of this study, it was evident that ineffective use of DOTS contributed to low TB cure rate, patients defaulting treatment and relapse. The recommendations focused on strengthening the effective use of DOTS. Another research that may focus on nurses caring for patients having TB in rural primary healthcare facilities of the North West Province could be necessary to obtain a broader picture of the use of DOTS.
